# Massive Osteolysis in a Modern Total Knee Prosthesis

**DOI:** 10.1155/2021/5507932

**Published:** 2021-10-20

**Authors:** Emmanuel Gibon, Jacquelyn A. Knapik, Hari K. Parvataneni

**Affiliations:** ^1^Division of Adult Reconstruction, Department of Orthopedics and Rehabilitation, University of Florida College of Medicine, University of Florida, Gainesville, FL, USA; ^2^Department of Pathology, Immunology and Laboratory Medicine, University of Florida College of Medicine, University of Florida, Gainesville, FL, USA

## Abstract

**Case:**

An 82-year-old woman underwent right total knee replacement with a sequentially irradiated and annealed highly cross-linked polyethylene insert. At 9 years, she was found to have a massive femoral osteolysis with an impending fracture.

**Conclusion:**

This case demonstrates a rare occurrence of massive femoral osteolysis, requiring revision surgery, with a sequentially irradiated and annealed highly cross-linked polyethylene.

## 1. Introduction

Total knee arthroplasty is a highly reliable and reproducible procedure to treat end-stage knee osteoarthritis. The Australian registry shows a survivorship of 91.4% at 18 years [1].

First-generation highly cross-linked polyethylene (HXLPE) was first introduced clinically in 2001 for total knee arthroplasty (TKA) [2]. However, due to concern for the potential risk of early fracture, its implementation has been more gradual than for total hip arthroplasty (THA). There remain concerns about transitioning from UHMWPE to HXLPE due to the reduction in ductility and fracture resistance associated with cross-linking. Nevertheless, in vitro studies have shown significant reduction of wear with first-generation HXLPE tibial inserts [3–5]. HXLPE tibial inserts are now predominantly used in Australia and represents 64.2% of all the PE inserts [1].

The X3® (Stryker, Mahwah, NJ) is a second-generation HXLPE, which was widely introduced in 2005. It is produced by gamma irradiating compression-molded GUR 1020 with 30 kGy of gamma irradiation followed by annealing to 130°C. These steps are repeated three times, and then, gas plasma sterilization occurs. The rationale behind this manufacturing technique for X3® was to remove enough free radicals to provide oxidation resistance while retaining the advantage of the UHMWPE mechanical properties. Wang et al. showed this process to be effective at dramatically eliminating free radicals equivalent to the level of virgin UHMWPE [6]. Accelerated aging studies showed similar results [7]. Knee simulator investigations reported wear reduction ranging from 64% to 86% compared to conventional UHMWPE [8–10]. Registry data shows a survivorship of the Triathlon® with X3® of 96.2% at 10 years [1]. Meneghini et al. [11] reported no radiographic osteolysis or mechanical failure of the tibial polyethylene at 5-year follow-up. Minoda et al. [12] showed a 58% reduction of the number of in vivo X3® wear particles compared to conventional PE in TKA.

Here, we present a case of catastrophic femoral osteolysis following a TKA with sequentially annealed highly cross-linked polyethylene, which is, to the best of our knowledge, the first report of this mode of failure in this modern design. The patient was informed that data concerning the case would be submitted for publication, and she provided consent.

## 2. Case Report

An 82-year-old woman presented to our institution for evaluation of her right TKA, which was performed in 2009 at an outside hospital. Her past medical history was significant only for rheumatoid arthritis (RA) for which she usually took adalimumab biweekly, methothrexate and celecoxib but reported this was well controlled.

Per her outside operative records, implants used at the initial surgery were a cemented Triathlon® CR femoral component, a 9 mm size 3 X3® CS insert, a size 3 Triathlon® universal tibial baseplate, and a 10 mm asymmetric Triathlon® X3® patella.

**Figure 1:**
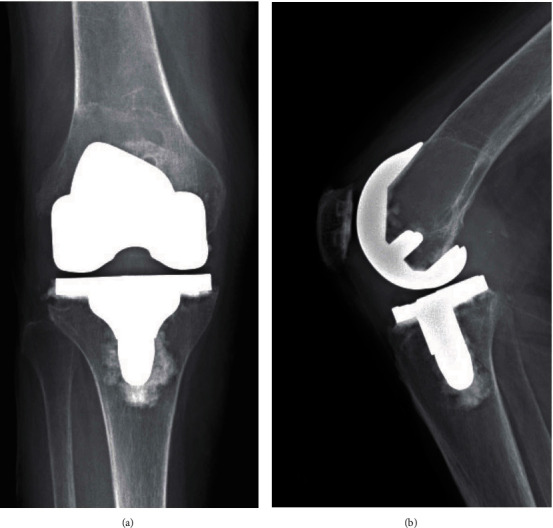
Right knee: (a) AP and (b) lateral radiographs at the time of presentation (February 2019).

The patient was initially referred by the primary surgeon to our oncology clinic in February 2019 due to findings of a distal femur lesion in her right knee discovered on follow-up X-rays (Figure 1) and better seen in a noncontrast MRI. At that time, the patient was pain-free but reported feelings of instability. The physical exam showed a well-healed midline surgical incision for TKA without complication. No obvious instability with varus/varus testing or drawer testing was found.

**Figure 2:**
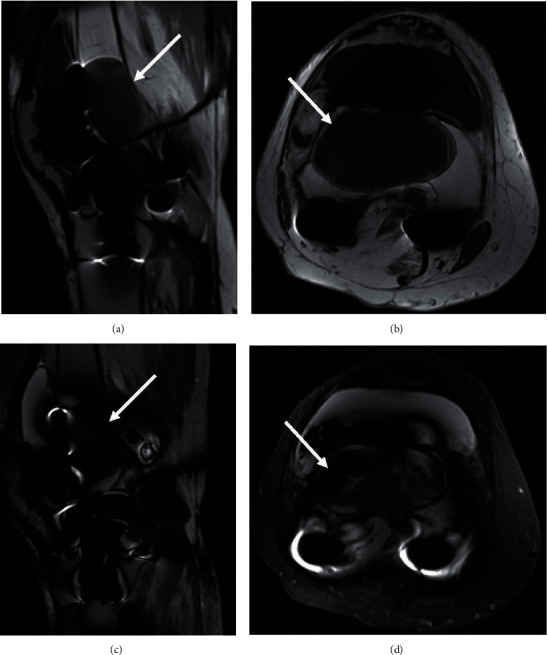
MRI of the right knee with IV gadolinium. In the distal femur, there is low T1 signal in the sagittal (a) and axial (b) views and heterogeneously predominantly low T2 signal in the sagittal (c) and axial (d) views with a sharp rim and thin peripheral enhancement (white arrows) that is in continuity with the prosthesis and typical for osteolysis secondary to particle disease (MRI machine: Titan 3T (Toshiba)).

MRI with IV contrast was performed to further characterize this mass in the right distal femur and rule out a malignant lesion (Figure 2). The MRI report showed “postsurgical changes of right TKA with findings typical for osteolysis secondary to particle disease and knee joint effusion with synovitis.” The orthopedic oncologist then referred the patient to our adult reconstruction clinic with very low suspicion for a neoplastic process.

**Figure 3:**
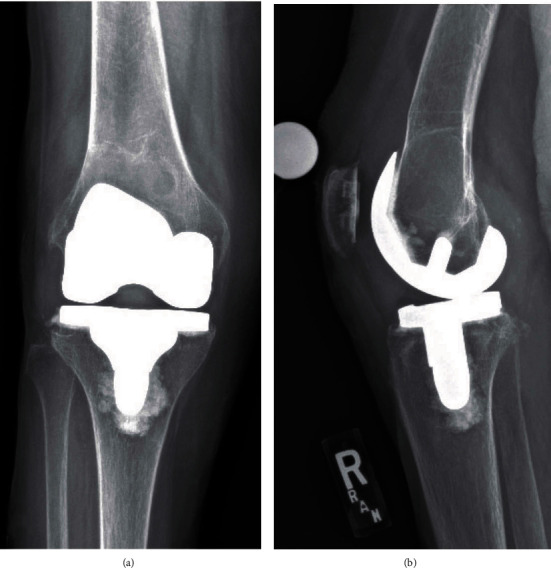
Right knee: (a) AP and (b) lateral radiographs six months later (October 2019).

The patient was assessed again in April 2019 and mentions occasional knee buckling but without pain. Her X-rays showed a focal well-circumscribed lucent lesion with thin geographic mineralized margin along the distal diametaphysis and deep to the cemented femoral component. There was thinning and weakening of the cortices in several areas. At that time, we recommended surgery due to the fracture risk. The patient declined but was agreeable to close surveillance. She was assessed two months later and did not want to consider surgery, again and refused X-rays due to absence of pain. She returned back a month later with mild knee pain and opted to proceed with a revision TKA for the diagnosis of massive femoral osteolysis and impending femoral fracture. The patient was later seen preoperatively, and the pain and X-rays remained stable (Figure 3).

**Figure 4:**
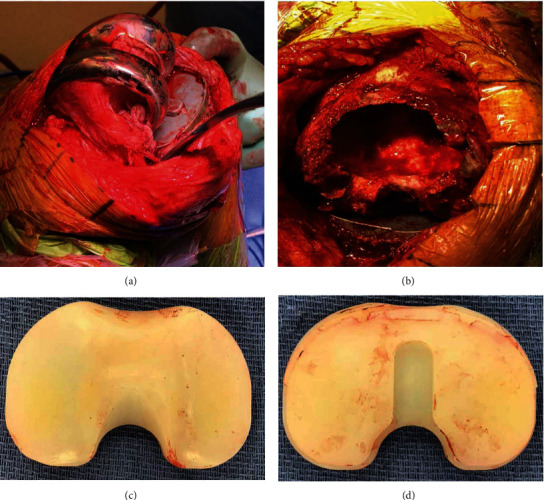
Intraoperative findings. (a) Lateral view of the distal femur showing massive osteolysis of the lateral condyle. (b) Top-down view of the distal femur after implant removal showing extension of the osteolysis into the canal. (c) Top view and (d) backside view of the X3® insert with no evidence of wear or surface damage. The entire polyethylene surface demonstrated yellow discoloration consistent with oxidation.

We utilized the prior incision and entered the join through a medial parapatellar arthrotomy. Clear synovial fluid was seen and sent for culture. The patella and tibia were mobilized and found to be well fixed, although the tibia had some osteolysis. The polyethylene was found to have minor oxidation, but no significant wear was found. We found no third bodies or other debris. We removed the insert and found no backside wear or any other explanation for this massive osteolysis. There was visual appearance of diffuse in vivo oxidation. Based on the extent of the osteolysis, we decided to revise the femur with a stem to bypass the defect. The femoral component was then removed with minimal bone loss. There were massive cystic changes with some debris that we sent for pathology (Figure 4). After refreshing the cuts, we proceeded with reconstruction. The femoral reconstruction was performed using a size A Tritanium® metaphyseal cone and Triathlon® femoral component with a 15 mm × 100 mm cemented stem extension and a 16 mm posterior stabilized X3® insert.

**Figure 5:**
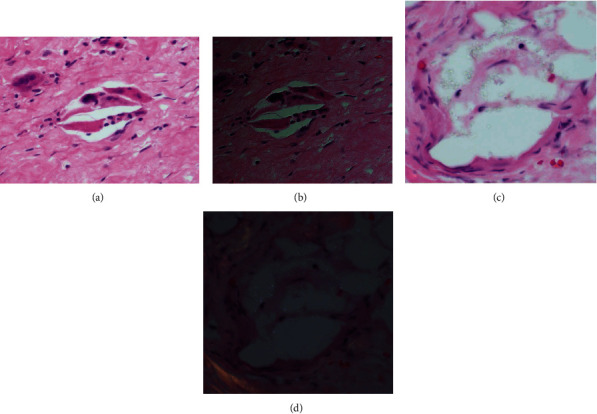
Histological analysis. (a, c) Multinucleated foreign body giant cells with engulfed shards of PE debris (H&E 40x). (b, d) H&E under refractile light.

All the cultures resulted negative. Microscopic evaluation (Figure 5) of the periprosthetic tissue revealed features typically observed when implant failure has taken place. Numerous multinucleated foreign body giant cells can be seen filled with debris from breakdown products. Some of the giant cells had empty spaces where engulfed methyl methacrylate cement was present but dissolved during tissue processing. Other foreign body giant cells contained shards of refractile debris and fragmented refractile particles. Macrophages also infiltrated the tissue and are filled with phagocytosed microparticles. Chronic inflammatory cells were present but there was no acute inflammation supporting that this was an aseptic process.

**Figure 6:**
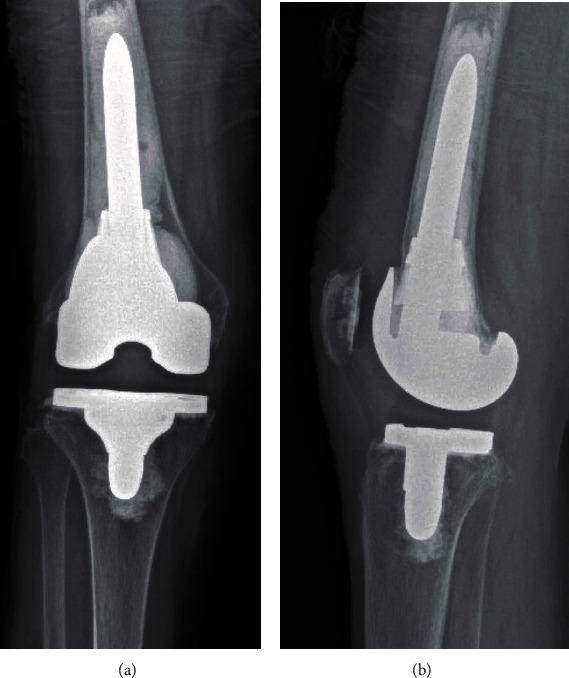
Right knee: (a) AP and (b) lateral radiographs at one-year follow-up.

At one-year clinical follow-up, the patient was noted to be doing well with no changes in her radiographic examination (Figure 6). Her incision healed uneventfully. The patient was pain free and ambulating without walking aids. At the last follow-up, her right knee range of motion was 0-130°.

## 3. Discussion

Osteolysis is now an uncommon reason for late failure of modern TKA and has been shown to represent 9% [13]. Our case is unique because it is the first report of a catastrophic femoral osteolysis at just under 10 years in an otherwise well-functioning TKA using a modern HXLPE insert which has an excellent track record. There are only a very limited number of case reports of previous failure of the X3® in TKA, and the failure modes were different than this report. Sonn and Meneghini [14] discussed an early failure of X3® at 5 years. The mechanism of failure was a completely worn through polyethylene. Kop et al. [15] investigated 15 implants from revision surgeries at a mean of 3 years. Of note, none of the revision was performed for isolated osteolysis. Furthermore, 4 of the 15 bearings exhibited signs of oxidation. MacDonald et al. [16] assessed the in vivo performance of X3® retrieved tibial inserts (*n* = 345) and compared them to gamma inert sterilized PE tibial inserts. The authors found that the X3® inserts had similar oxidation levels when compared to gamma inert controls. Moreover, they had 6 cases of posterior fracture in the X3® cohort and none in the control group.

In our case, one could propose that RA could have resulted in the bony changes. However, the patient's disease was well controlled and the pathology analysis did not notice any microscopic tissue modification pointing toward RA. Pathology also ruled out a neoplasm. Polyethylene-induced osteolysis was further confirmed by histological analysis. Examination of our retrieval PE insert showed signs of burnishing, which is not an uncommon finding for tibial bearings. We did not observe any fracture or delamination of the implant but significant oxidation was seen.

Typical risk factors for osteolysis associated with TKA are malalignment of the mechanical axis and improper ligament balancing, early-generation PE sterilization techniques, back-side wear, third-body abrasion, young age, increased activity level, and elevated body mass index. In our case, the patient was a low-demand 82-year-old woman with a BMI of 23.8 kg/m^2^. In this present case, one potential explanation could be an isolated issue with a batch from manufacturing since this is such an unusual case.

The Manufacturer and User Facility Device Experience (MAUDE) database captures failure patterns with certain FDA cleared medical devices in a timely manner. We reviewed this database between 2005 and 2020 and found 15 reported cases related to the X3® insert, and 10 of them were about the tibial inserts. None of them were related to osteolysis.

The use of HXLPE in TKA is still controversial. A recent study by Partridge et al. [17] that combined registries from England, Wales, and Northern Ireland showed that conventional polyethylene had significantly lower aseptic revision rates than HXLPE, except for a very specific subset of patients.

## 4. Conclusion

In summary, this case demonstrates that primary TKA performed with sequentially irradiated and annealed highly cross-linked polyethylene are not immune to polyethylene-induced osteolysis. Although X3® shows excellent survivorship at 10 years for TKA, routine surveillance of patients might be warranted especially around the first decade after implantation.

## Data Availability

The data used to support the findings of this study are included within the article.
